# Author Correction: Self-utility distance as a computational approach to understanding self-concept clarity

**DOI:** 10.1038/s44271-025-00244-3

**Published:** 2025-04-11

**Authors:** Josué García-Arch, Christoph W. Korn, Lluís Fuentemilla

**Affiliations:** 1 Department of Cognition, Development and Education Psychology, Faculty of Psychology, University of Barcelona, Barcelona, Spain; 2 Institute of Neuroscience (UBNeuro), University of Barcelona, Barcelona, Spain; 3 Section Social Neuroscience, Department of General Psychiatry, University of Heidelberg, Heidelberg, Germany; 4 Bellvitge Institute for Biomedical Research, 08908 Hospitalet de Llobregat, Barcelona, Spain

**Keywords:** Human behaviour, Psychology

Correction to: *Communications Psychology* 10.1038/s44271-025-00231-8, published online 25 March 2025

The original version of this article omitted the following from the Acknowledgements: “Funded by AGAUR 2021 SGR 00352”. This has now been corrected in both the PDF and HTML versions of the article.

