# Supplementary material for: Self-utility distance as a computational approach to understanding self-concept clarity
Source: Commun Psychol. 2025 Mar 25;3:50. doi: 10.1038/s44271-025-00231-8 (PMC11937342; doi:10.1038/s44271-025-00231-8)
Supplement: Supplementary file 2 — Supplementary Information [file 44271_2025_231_MOESM2_ESM.pdf]

Supplementary Table S1. Stimuli

**Positive adjectives**	**Negative adjectives**
Adventurous	Absent-minded
Clever	Anxious
Constant	Arrogant
Convincing	Authoritarian
Cooperative	Capricious
Cordial	Disorganized
Cultured	Distant
Detail-oriented	Exaggerated
Discreet	Forgetful
Energetic	Fussy
Faithful	Gullible
Friendly	Hot-tempered
Humble	Impulsive
Interesting	Indecisive
Intuitive	Irritable
Meticulous	Materialistic
Modest	Nervous
Original	Noisy
Peaceful	Obsessive
Persevering	Sarcastic
Practical	Self-centered
Realistic	Shy
Sincere	Solitary
Sociable	Stubborn
Tolerant	Suggestible

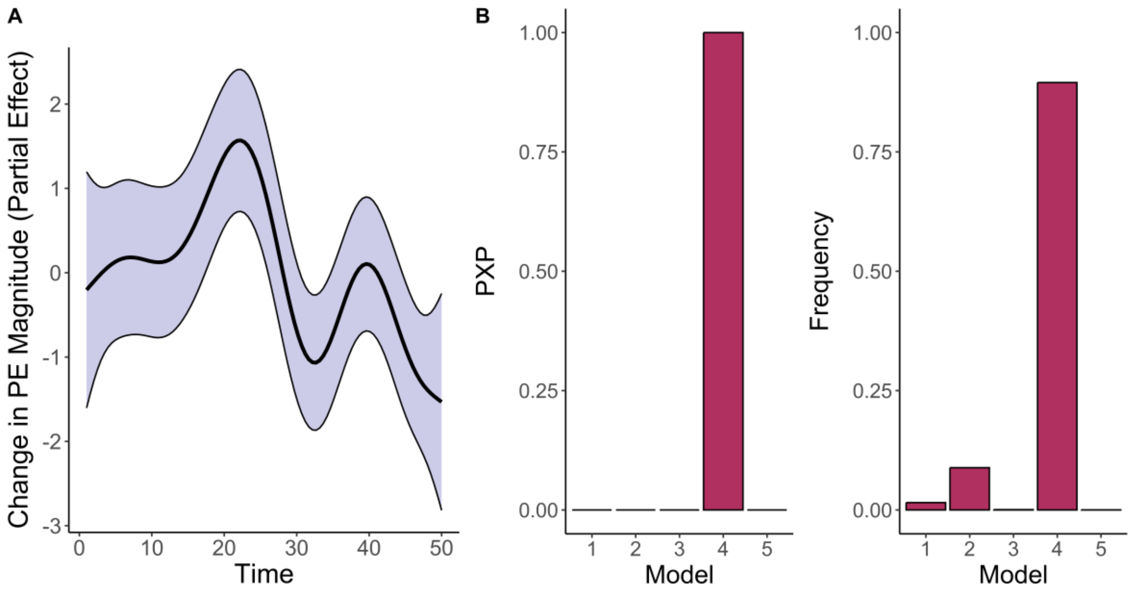

Supplementary Figure S1. A. Results from generalized additive model predicting prediction errors (PE) with time (trial number), the light blue shaded region represents the 95% confidence interval for the regression. B. The first plot (left) depicts the Protected Exceedance Probability, a robust index that computes the probability that one model is the most frequent model in the model space, accounting for the possibility that differences in model evidence are due to chance. The second plot (right) depicts model's frequency across participants.

Supplementary Note 1. External feedback ratings and similarity matrix.

Following prior research (Frolichs et al., 2022) we recruited independent samples to obtain (real) feedback ratings as well as to compute the similarity matrices needed for our computational models. We recruited a sample of 250 individuals of similar age and same educational background (psychology undergraduates) as the sample of our study. 18 participants were excluded because of missing more than 20% of the responses. Similar to study 1, participants were asked to evaluate the utility of the different 50 traits in the list (Table S1). The order of presentation was randomized. We computed feedback ratings (averages for each trait among participants) and similarity matrices (pearson correlations) from the resulting data.

Supplementary Note 2. Parameter recovery

We tested the robustness of our computational models by conducting parameter recovery, a method used to determine how well models can recover (i.e., estimate) a priori known parameter values. We conducted the simulation of two hundred datasets with randomly sampled parameters and noise added in the last step. Parameter recovery evaluated models' capacity in finding the true underlying mechanisms that generated the data (known input parameters) with those estimated after model fitting (recovered parameters). Correlations between all input and recovered parameters of all models are reported in Figure S2. We also constructed of a confusion matrix, which assessed the distinguishability of our models. That is, each model's capacity to be accurately identified from the data it generated. We employed exceedance probability to quantify the likelihood that each model was the best fit for the data it had generated, and for the data generated by the rest of models. Results yielded an identity matrix, where all exceedance probabilities for the data-generating models hit 1, with all others at 0, demonstrating robust model distinguishability.

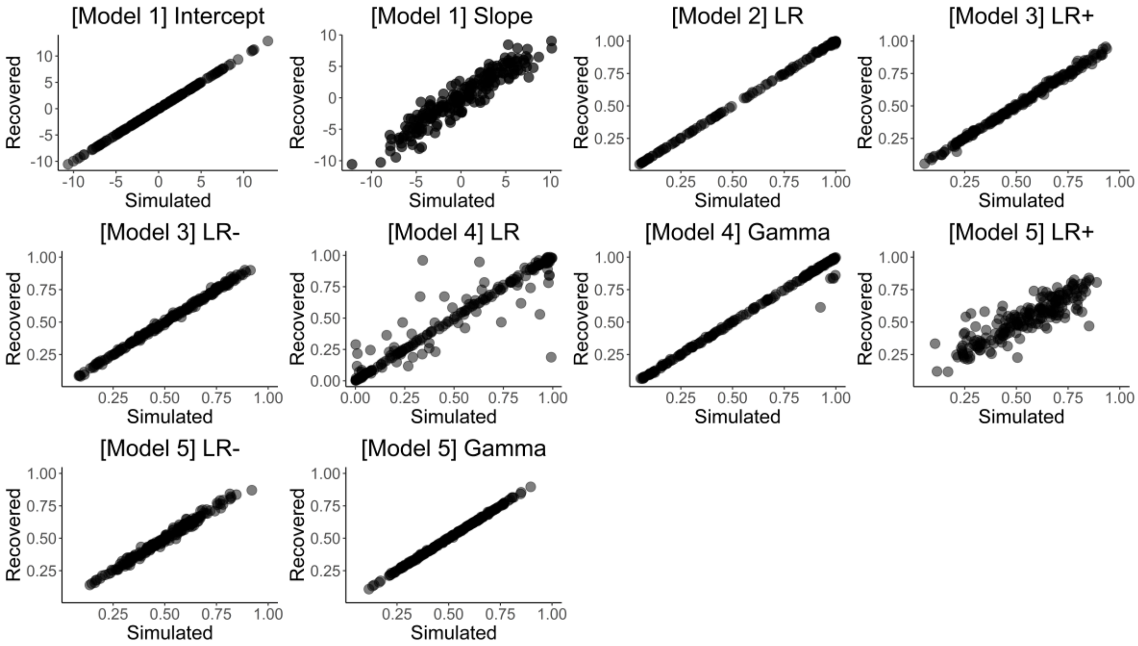

Supplementary Figure S2. Parameter Recovery. Relationship between simulated and recovered parameters (x and y axis, respectively). All correlations between recovered and simulated parameters were satisfactory (*all r* > .87).

Supplementary Note 3. Additional analysys.

To test whether initial self-ratings merely serve as a starting point or exert a sustained, motivational influence on the learning process, we re-estimated Models 2 and 3 using each participant's own self-ratings as the initial trait utility values. Using the same Hierarchical Bayesian Inference (HBI) procedure, we found that the model comparisons remained robust, with Model 4 still emerging as the winning model (model frequency = 91.5%, Protected Exceedance Probability = 1). This result suggests that the initial self-ratings persistently influence the updating process, reinforcing the interpretation that individuals are motivated to align new feedback with their established self-concept.
